# Slowly resorbable biosynthetic mesh: 2-year results in VHWG grade 3 hernia repair

**DOI:** 10.1007/s10029-021-02453-1

**Published:** 2021-07-19

**Authors:** M. M. J. Van Rooijen, T. Tollens, L. N. Jørgensen, T. S. de Vries Reilingh, G. Piessen, F. Köckerling, M. Miserez, A. C. J. Windsor, F. Berrevoet, R. H. Fortelny, B. Dousset, G. Woeste, H. L. van Westreenen, F. Gossetti, J. F. Lange, G. W. M. Tetteroo, A. Koch, J. Jeekel

**Affiliations:** 1 Department of Surgery, Erasmus University Medical Centre Rotterdam, Room Ee-1453, PO BOX 2040, 3000 CA Rotterdam, The Netherlands; 2 Department of General Surgery, Imelda Hospital, Bonheiden, Belgium; 3 Department of Surgery, Bispebjerg Hospital, University of Copenhagen, Copenhagen, Denmark; 4 Department of Surgery, Elkerliek Hospital, Helmond, The Netherlands; 5 Department of Surgery, University Hospital Lille, Lille, France; 6 Department of Surgery, Vivantes Klinikum Spandau, Berlin, Germany; 7 Department of Abdominal Surgery, University Hospital Leuven, Leuven, Belgium; 8 Department of Colorectal Surgery, University College London Hospital, London, UK; 9 Department of General and Hepatobiliary Surgery, University Hospital Ghent, Ghent, Belgium; 10 Department of General, Visceral and Oncologic Surgery, Wilhelminen Hospital, Vienna, Austria; 11 Department of Digestive, Hepatobiliary and Endocrine Surgery, Hôpital Cochin, Paris, France; 12 Klinikum der Johann Wolfgang Goethe-Universität, Frankfurt am Main, Germany; 13 Department of Surgery, Isala Zwolle, Zwolle, The Netherlands; 14 Università di Roma Sapienza, Rome, Italy; 15 Department of Surgery, IJsselland Ziekenhuis, Capelle aan den Ijssel, The Netherlands; 16 Chirurgische Praxis Cottbus, Cottbus, Germany; 17 Department of Neuroscience, Erasmus University Medical Centre Rotterdam, Rotterdam, The Netherlands

**Keywords:** Biosynthetic mesh, Incisional hernia, Hernia recurrence, Hernia surgery

## Abstract

**Introduction:**

Information on the long-term performance of biosynthetic meshes is scarce. This study analyses the performance of biosynthetic mesh (Phasix™) over 24 months.

**Methods:**

A prospective, international European multi-center trial is described. Adult patients with a Ventral Hernia Working Group (VHWG) grade 3 incisional hernia larger than 10 cm^2^, scheduled for elective repair, were included. Biosynthetic mesh was placed in sublay position. Short-term outcomes included 3-month surgical site occurrences (SSO), and long-term outcomes comprised hernia recurrence, reoperation, and quality of life assessments until 24 months.

**Results:**

Eighty-four patients were treated with biosynthetic mesh. Twenty-two patients (26.2%) developed 34 SSOs, of which 32 occurred within 3 months (primary endpoint). Eight patients (11.0%) developed a hernia recurrence. In 13 patients (15.5%), 14 reoperations took place, of which 6 were performed for hernia recurrence (42.9%), 3 for mesh infection (21.4%), and in 7 of which the mesh was explanted (50%). Compared to baseline, quality of life outcomes showed no significant difference after 24 months. Despite theoretical resorption, 10.7% of patients reported presence of mesh sensation in daily life 24 months after surgery.

**Conclusion:**

After 2 years of follow-up, hernia repair with biosynthetic mesh shows manageable SSO rates and favorable recurrence rates in VHWG grade 3 patients. No statistically significant improvement in quality of life or reduction of pain was observed. Few patients report lasting presence of mesh sensation. Results of biosynthetic mesh after longer periods of follow-up on recurrences and remodeling will provide further valuable information to make clear recommendations.

**Trial registration:**

Registered on clinicaltrials.gov (NCT02720042), March 25, 2016.

## Introduction

Incisional hernias occur in up to 20% of patients after midline laparotomy [1]. In case of complaints, such as pain and reduced quality of life (QoL), operative repair is indicated [2]. This repair traditionally took place with permanent synthetic meshes, as these have proven to reduce the risk of recurrence compared to primary closure [1, 3–5]. However, permanent synthetic meshes remain in the body as foreign material, increasing the risk of seromas, infections, enterocutaneous fistulas, and chronic pain [3, 6, 7]. This has led to the development of resorbable biologic meshes, which would not cause foreign material to be left in the body, and which were hypothesized to be more infection resistant [8].

However, resorbable biologic meshes have faced problems in resorption rate; too quick resorption does not support abdominal wall regeneration and will consequently lead to higher recurrence rates. To tackle the problem of too rapid resorption, slowly resorbable biosynthetic meshes have been developed recently for the field of abdominal wall reconstruction [9], among which products made from poly-4-hydroxybutyrate (P4HB). Due to the resorption of these products, no foreign material remains behind, yet this slow resorption process is only essentially complete after 12–18 months, providing initial mechanical strength comparable to polypropylene mesh to support the native abdominal wall for cellular ingrowth and remodelling [10, 11]. However, most existing knowledge on these P4HB products is based on in vitro experiments and animal models, and sufficient clinical data is lacking. Hypothetically, the gradual mesh resorption would allow natural forces to put gradual strain on the abdominal wall muscles and aponeuroses, which could restore and reshape the functional tissue structure. This “use it or lose it” concept is known from the remodelling of bone, and might also be applicable to the abdominal wall during recovery after hernia repair [12, 13]. All above described characteristics of P4HB meshes are especially desirable in (potentially) contaminated surgical fields, such as Ventral Hernia Working Group (VHWG) grade 3 hernias [14].

Currently, research is focused on whether these biosynthetic meshes live up to their expectations, as they are more costly than synthetic meshes. Our early results suggest that these meshes perform comparable to synthetic mesh on the short-term in high-risk patients [15]. However, information on long-term performance is scarce [16]. It has yet to be discovered whether biosynthetic meshes do indeed cause adequate abdominal wall remodelling and cause less incisional hernias to recur, especially in a high-risk population. Therefore, the objective of this study was to collect additional data on the long-term performance of P4HB mesh in patients requiring VHWG grade 3 hernia repair.

## Methods

### Study design

We conducted this prospective single-arm trial in 15 European hospitals. Adults scheduled for elective VHWG grade 3 incisional hernia repair were included. VHWG grade 3 comprises hernias in a surgical site in which there has been previous wound infection, on which a stoma is present, or in which violation of the gastro-intestinal tract takes place. Additional inclusion criteria with regard to the hernia were a midline position and a size of more than 10 cm^2^. An elaborate overview of the exclusion criteria has been previously published [15, 17].

The research protocol was approved by the institutional review board or health authority of every participating center, has been previously published [17], and is registered at clinicaltrials.gov (NCT02720042). All included patients gave written informed consent prior to any study procedure.

### Procedures

Final eligibility of patients was determined during surgery, after which open ventral hernia repair was performed. A P4HB biosynthetic mesh (Phasix™ Mesh, C.R. Bard, Inc., Warwick, RI) was placed retro-rectus as fascia augmentation, overlapping all edges of the defect by 5 cm. When this could not be achieved, onlay placement was allowed. Component separation techniques (CST) were used when considered appropriate by the surgeon. The mesh was fixated with slowly resorbable sutures at 5–6-centimeter intervals.

Postoperatively, patients received the standard care of their treating center. Follow-up took place after 1, 3, 6, 12, 18, and 24 months, during which patients underwent physical examination by a medical doctor, and were asked to fill out QoL questionnaires: Carolinas Comfort Scale (CCS) [18], EQ-5D [19], and Visual Analog Scale (VAS). The CCS measures severity of mesh sensation and pain with a score from 0 (no complaints) to 5 (disabling symptoms). The EQ-5D assesses 5 dimensions of health (mobility, self-care, usual activities, pain or discomfort, and anxiety or depression) and allows patients to rate their “Health Today” on a scale from 0 to 100 [19]. Pain was assessed through the VAS, anchored by “no pain” (score 0) and “worst imaginable pain” (score 10) on a 10-cm scale. Patients were censored in case of death or when they ended follow-up.

### Outcomes

The short-term, primary outcome was surgical site occurrence (SSO) up to 3 months after surgery that required medical or surgical intervention. Hematoma, seroma, surgical site infection (SSI) [20], wound dehiscence, skin necrosis, and fistula were all considered SSOs. Additionally, the surgical procedure time, the length of hospital stay, and the time to return to work were outcomes of interest.

The long-term, secondary outcome was the recurrence rate determined per physical examination, and if uncertain, per ultrasound examination, CT scan, or MRI. Other long-term outcomes of interest were reoperation rate, SSO rate up to 24 months, and QoL and pain outcomes (CCS, VAS, and EQ-5D).

### Sample size and statistical analysis

Seventy-five patients were deemed sufficient to evaluate the performance of Phasix™ Mesh. Anticipating an attrition rate of 10%, the aim was to include 85 patients. Data from all included patients implanted with Phasix™ Mesh were analyzed. Baseline characteristics were summarized with frequency counts and percentages, or with mean and standard deviation (sd). Follow-up is summarized through median with range. Short-term and long-term endpoints are reported as proportions or means with a 95% confidence interval (95% CI) or as medians with range or interquartile range (IQR). The long-term endpoint of recurrence was assessed through time-to-event analysis. No missing value imputation methods were used. Analysis was through an intention-to-treat principle, with Statistical Analysis System (SAS), Version 9.3 and R version 3.3.3.

## Results

Between March 2016 and April 2017, 84 patients were included for analysis. Patient and hernia characteristics are presented in Table 1. The median follow-up was 24 months (734 days; range 9–834 days).

**Table 1:** Baseline patient and surgical characteristics presented as *n* (%) or mean (sd)

	*N* = 84
*Patient characteristics*	
Gender (%)	
Male	51 (60.7)
Female	33 (39.3)
Age, years (sd)	62.5 (12.4)
BMI, kg/m^2^ (sd)	27.8 (4.0)
Obesity (BMI > 30 kg/m^2^) (%)	22 (26.2)
Hypertension (%)	39 (46.4)
Smoking (%)	39 (46.4)
Lung disease (%)	19 (22.6)
Diabetes (%)	12 (14.3)
Corticosteroid use (%)	3 (3.6)
Cancer history (%)	35 (41.7)
Chronic pain (%)	10 (11.9)
Reason VHWG 3 (%)	
Previous wound infection	49 (58.3)
Stoma present	16 (19.0)
Creation of a stoma	5 (6.0)
Violation of the GI tract	1 (1.2)
Combination of above	8 (9.5)
Other	5 (6.0)
*Surgical characteristics*	
Hernia defect (sd)	
Length (cm)	12.1 (5.7)
Width (cm)	8.0 (3.5)
Area (cm^2^)	109.2 (87.9)
Incisional hernia (%)	
Primary incisional hernia	68 (81.0)
First recurrence	9 (10.7)
> 1 recurrence	7 (8.4)
Explant of previous mesh (%)	10 (11.9)
Use of CST (%)	49 (58.3)

*BMI* body mass index, *sd* standard deviation, *CST* component separation technique

### Short-term outcomes

Within 3 months, 22 patients (26.2%; 95%CI: 17.2–36.9%) had developed 32 SSOs. Four patients with SSOs required hospitalization (12.5%) and three patients required surgical intervention (9.4%). The majority—11 (34.4%)—of these SSOs were SSIs. All but one SSI arose within the first month after surgery. An extended overview of the short-term outcomes has been published previously [15].

The median surgical procedure time from incision to closure was 163 min (range 60–696 min). After surgery, the median length of stay was 8 days (range 3–38 days). Twenty patients (23.8%) spent part of their hospital stay in the intensive care unit (ICU), with a maximum duration of 18 days (median 2.0 days, IQR 1–4 days). Of the 26 patients that were employed during the study, the median number of days for returning to work was 55 (range 7–785 days).

### Long-term outcomes

**Fig. 1:**
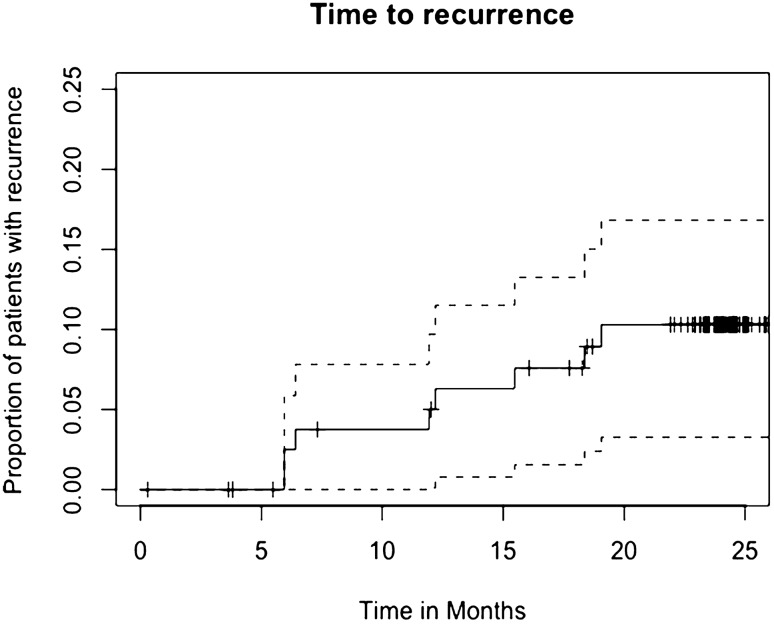
Hernia recurrences over time (solid line) with 95% confidence interval (dotted lines). Censored subjects are marked with “ + ”

Over the course of 24 months, 15 patients (17.9%) did not complete their 24-month follow-up visit, due to being incapacitated or lost to follow-up (*n* = 7), death (*n* = 3), withdrew from participation (*n* = 1), or had the biosynthetic mesh explanted (*n* = 4; of which 3 due to recurrence). Of these 15 patients, 4 experienced a hernia recurrence. An additional four recurrences occurred in the group (*n* = 69) that did complete 24-month follow-up. Therefore, 8 patients out of a total of 73 (11.0%) developed a recurrence of their incisional hernia (95% CI 3.8–18.1%). The hernia recurrences and censored patients over time are depicted in Fig. 1.

In 13 patients of all 84 (15.5%; 95% CI 8.5–25.0%), 14 reoperations took place. Six were performed for hernia recurrence (42.9%, of which one with a concomitant mesh infection), three for mesh infection (21.4%), one each for subcutaneous hematoma, ileus, and subcutaneous abscess (each 7.1%), and two for other reasons (14.3%). During 7 of these 14 reoperations (50%), a full or partial explant of the biosynthetic mesh was deemed necessary. Of the reoperations for mesh infection, two mesh infections were secondary to active infection present at the time of index procedure (VHWG grade 4), and one infection was secondary to faecal peritonitis postoperatively.

With regard to the SSOs up to 24 months, only two more developed after the short-term results of 3 months. The division of the cumulative 34 SSOs is shown in Table 2. Of note—though not significant—19.6% of men experienced SSOs, compared to 36.4% of women.

**Table 2:** Surgical site occurrence (SSO) development within 24 months

	Total (*n* = 84)
Patients with SSO, *n* (%)	22 (26.2)
Total SSO	34
SSI	11
Wound dehiscence	9
Seroma	7
Hematoma	2
Skin necrosis	2
Fistula	3

*SSI* surgical site infection

**Fig. 2:**
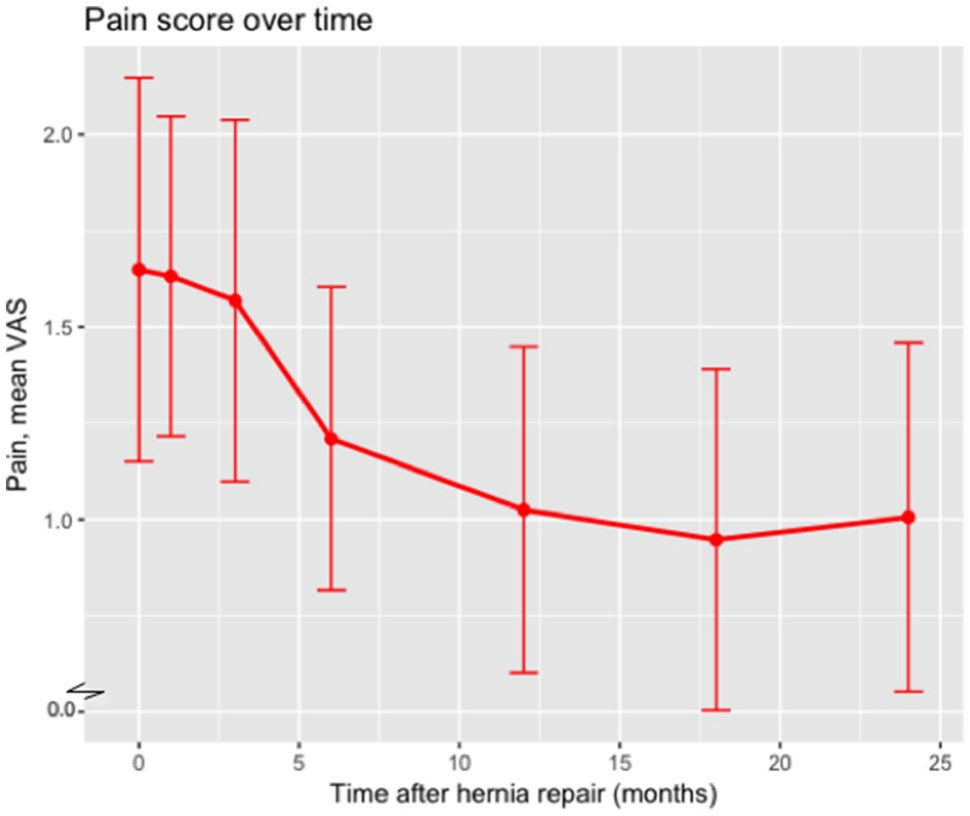
Mean VAS score for pain over time including 95% confidence intervals. *VAS* visual analog scale

**Fig. 3:**
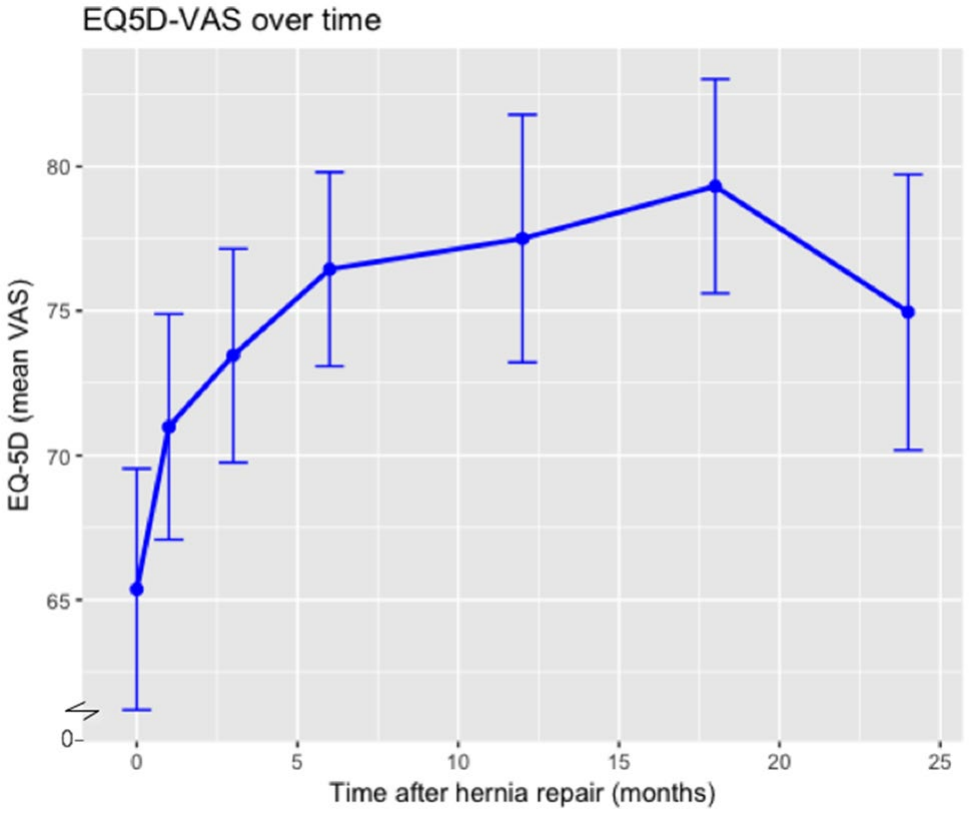
Mean EQ-5D VAS score (“Health Today”) over time, including 95% confidence intervals. EQ-5D VAS is a self-reported score on a scale from 0 to 100, with higher values representing a higher quality of life

With regard to the QoL measurements up to 24 months, the VAS score dropped 0.7 on average (sd: 2.45). The course of the mean VAS score over time is depicted in Fig. 2. The EQ-5D measure of “Health Today” is similarly depicted in Fig. 3.

Pertaining the health domains researched in the EQ-5D questionnaire, the proportion of patients reporting no problems in each domain increases over time. This is also shown in Table 3.

**Table 3:** The five dimensions of the EQ-5D questionnaire per time point

	Baseline	1 mo	3 mo	6 mo	12 mo	18 mo	24 mo
*n*	84	82	83	80	77	71	69
Mobility (%)	57 (68)	51 (62)	64 (77)	63 (79)	62 (81)	57 (80)	56 (81)
Self-care (%)	67 (80)	59 (72)	70 (84)	71 (89)	71 (92)	63 (89)	64 (93)
Usual activities (%)	48 (57)	41 (50)	60 (72)	65 (81)	61 (79)	56 (79)	55 (80)
No pain/discomfort (%)	37 (44)	37 (45)	47 (57)	45 (56)	51 (66)	44 (62)	47 (68)
No anxiety/depression (%)	56 (67)	60 (73)	59 (71)	63 (79)	61 (79)	61 (86)	56 (81)

Number and percentage of patients reporting no problems in mobility, self-care, or usual activities, and reporting no pain or no anxiety

*mo* month

**Fig. 4:**
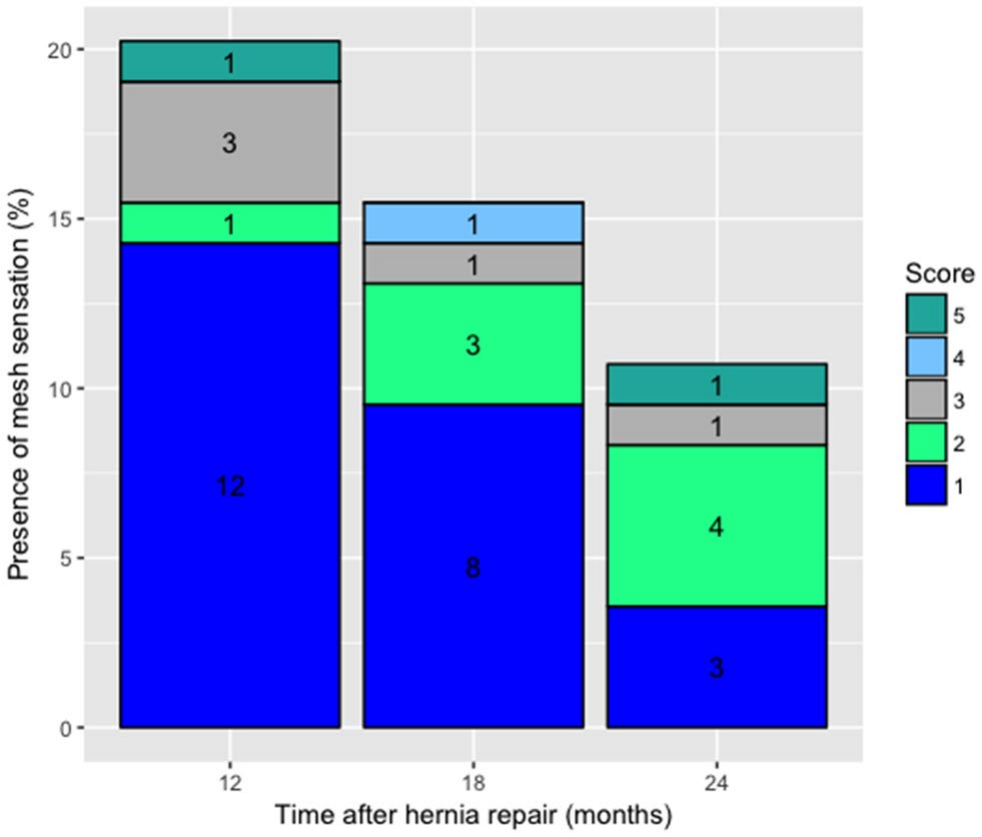
Patients reporting a sensation of the mesh during activities of daily living in the CCS questionnaire at timepoints at which the Phasix™ mesh should have been fully resorbed. Absolute numbers are depicted within the stacked bars. Score: 5 = disabling; 4 = severe; 3 = moderate and/or daily; 2 = bothersome but not daily; 1 = mild but not bothersome

Results of the CCS questionnaire can be viewed in Fig. 4. Proportions of patients reporting a sensation of the mesh during the activities of daily living (ADL) show a downward trend for the 12-month, 18-month and 24-month follow-up point. At these time points, the mesh is expected to have been fully resorbed by the body. At 12 months, 20.2% of patients still reports mesh sensation during ADL, and at 24 months, this feeling remains in nine patients. In comparison, 23 patients (27.4%) report to have sensation of the mesh during ADL one month postoperatively.

## Discussion

During 2 years of follow-up, 22 patients developed 34 SSOs (26.2%), and a recurrence rate of 11.0% was observed, demonstrating reasonably good results. Three reoperations were performed due to mesh infection (which were expected due to VHWG grade 4 and the development of faecal peritonitis after surgery). Other studies with P4HB mesh reported similar findings: in a small study of 25 patients treated with P4HB mesh for ventral or incisional hernias, 2 hernias recurred (8%), at 12 and 24 months, respectively [21]. In a prospective study of 121 VHWG grade 2 hernia patients, 11 patients (9%) had developed a recurrence after 18 months of follow-up [22]. Other authors found a 12.7% and 12.4% recurrence rate after the use of P4HB mesh, after 43.1 months and 11.7 months, respectively [23, 24].

Especially in researched high-risk patient group, the recurrence rate is relatively low in comparison with current literature. Reported recurrence rates in *VHWG grade 3 hernias* after an average follow-up of 12–28 months vary from 18.5% with permanent synthetic mesh [25], to around 16% with biologic mesh [26, 27], to 32.5% in a combined cohort of biologic and permanent synthetic mesh [28]. Recurrence rates of up to 25% do frequently occur [29]. A recent study showed equally positive results to ours with the use of permanent synthetic mesh *in contaminated fields*. In this retrospective analysis of 402 patients, 14.2% had an SSI and 10.5% had a recurrence after a median follow-up of 30 months [30]. However, comparison between studies is difficult, since many researched factors differ such as type of mesh, operative technique, study design (prospective or retrospective), and follow-up time. Nonetheless, 11% recurrence in this prospective study seems overall a good result.

Hypothetically, resorbable materials seem preferential over permanent meshes that remain in the body, as they might reduce fear and anxiety, and possibly reduce chronic pain development and sinus formation due to the occasionally described shrinkage of permanent synthetic mesh products [31]. Resorbable meshes might additionally prevent the risk of developing the (rare) complication of enterocutaneous fistula [32–34]. Additionally, due to being resorbed, biosynthetic meshes might possibly be more suitable in contaminated wound sites or high-risk patients. Despite studies tentatively suggesting this too [35], we found a 10.7% lasting sensation of mesh after 24 months, when the mesh is expected to be fully resorbed. This shows that further research is required to provide further elucidation.

Although we found comparable numbers to the COBRA study [36], the QoL scores over time showed a non-significant upward trend from baseline. These findings could have been positively biased over time, as censored patients are more likely to have worse scores. Although hernia repair can increase QoL on the long term [37, 38], QoL is affected short-term postoperatively; the majority of SSOs occurred within one month after surgery (91.2%) and more patients report physical problems at one month of follow-up (Table 3). This implicates that hernia repair surgery should be carefully considered in this high-risk patient group, also as nearly a quarter of patients had to spend some nights on the ICU after surgery.

The reoperation rate of 15.5% is relatively high. However, as shown through the presence of many comorbidities and the rate of patients admitted postoperatively to the ICU, this is most likely a high-risk patient group. Previous malignant disease, previous hernia repair, and bowel resection are risk factors for postoperative complications [39], and are ample present in this patient population. As two VHWG grade 4 patients were mistakenly included, and faecal peritonitis was developed after concomitant procedures in one patient, three reoperations due to mesh infection were not unexpected.

A remarkable finding is the presence of mesh sensation during ADL in 20.2% and 10.7% of patients after 12 and 24 months, respectively. At these time points, the P4HB biosynthetic mesh is expected to have been fully resorbed. Several explanations for these findings can be considered: either patients filled out the CCS questionnaire incorrectly; or patients had a strong belief they still felt mesh without it actually being there; or incomplete resorption or inadequate remodeling has taken place, in which scar tissue and adhesions cause a sensation of something “being there”. Unfortunately, the true cause of these findings cannot be unveiled.

### Limitations

Our study has some limitations. Multiple centers in multiple countries across Europe participated in the study, with inter-surgeon difference in hernia repair as a result. However, all centers are experienced hernia centers with abdominal wall specialists performing the surgery. Both affect the external validity: the multi-center design increases the external validity, yet “real-life” clinical results might be somewhat less as only specialists were involved.

The use of questionnaires is also associated with limitations. Despite widespread popularity of the used (translated) questionnaires, these have not been validated in all languages. Especially in translated instruments, the question or intention of the instrument can be unclear for patients, resulting in erroneous completion of the questionnaire. Moreover, this is an international study, in which culture can have influenced patients’ reporting; this can possibly minimize comparability between answers, and it stresses the subjectivity of questionnaires.

## Implications and conclusion

Overall, P4HB biosynthetic mesh is feasible for use in hernia repair, and, although three reoperations had to be performed due to mesh infection, results in a favorable recurrence rate up to 2 years after surgery. However, further research is desirable into the performance of the biosynthetic mesh over an even longer period of time. Present study focused on the clinical applicability and outcomes of P4HB mesh, but further research with radiological measurements is warranted to answer remaining questions on long-term recurrence and abdominal wall remodeling, especially with our findings of persisting mesh sensation in mind.

Although not assessed in present study, cost–benefit analyses should also be conducted. P4HB mesh is often 2.5 × more expensive than traditional permanent synthetic mesh, but one study indicates that its use might reduce healthcare costs with approximately 770 euros per incisional hernia repair [40]. However, more research on (long-term) clinical outcomes, reoperations, and work incapacity should be conducted to assess cost–utility.

In conclusion, P4HB biosynthetic mesh use is feasible for incisional hernia repair with regard to SSOs and infection rate when performed by experienced hernia specialists, and results in a recurrence rate of 11.0% after 2 years in potentially contaminated hernia sites. Longer follow-up data on abdominal wall remodeling and recurrences are needed to draw definite conclusions on the use of P4HB mesh.

## Data Availability

Not applicable.
